# Association of multiple mineral and vitamin B group intake with blood glucose using quantile regression analysis: NHANES 2007–2014

**DOI:** 10.29219/fnr.v63.3560

**Published:** 2019-12-03

**Authors:** Shoumeng Yan, Meng Li, Xiaoyu Ma, Shan Jiang, Mengzi Sun, Changcong Wang, Yingan Pan, Chong Sun, Yan Yao, Lina Jin, Bo Li

**Affiliations:** 1 Department of Epidemiology and Biostatistics, School of Public Health, Jilin University, Changchun, P. R. China; 2 Key Laboratory of Organ Regeneration and Transplantation of Ministry of Education, School of Public Health, Jilin University, Changchun, Jilin, P. R. China

**Keywords:** hyperglycaemia, quantile regression, vitamin B, mineral, blood glucose

## Abstract

**Background:**

Hyperglycaemia and diabetes have become major public health problems worldwide. There is increasing evidence that minerals and the vitamin B group might play specific roles in hyperglycaemia and the pathogenesis and progression of diabetes or metabolic complications.

**Objectives:**

The main aim of this study is to investigate the effect of mineral and vitamin B group supplementation on the blood glucose levels of different populations.

**Design:**

This was a cross-sectional study. Data from the National Health and Nutrition Examination Survey (NHANES) 2007–2014 were used in this study. A total of 8,322 participants (4,169 men and 4,153 women) were included in the study. Quantile regression (QR) was performed to identify the influence of mineral and vitamin B group intake on the level of fasting plasma glucose (FPG) in individuals in different quantiles of FPG.

**Results:**

After adjusting for age, income, education, race, smoking, and alcohol consumption, FPG had a negative association with folic acid in individuals with normal or high FPG, with calcium in individuals with normal FPG, and with magnesium in males. FPG was negatively associated with folic acid and calcium in individuals with normal FPG, and magnesium in most of the quantiles for females.

**Discussion:**

Hyperglycaemia and diabetes are currently becoming popular research topics. However, little is known about how the whole continuum of blood glucose is associated with commonly researched nutrient supplementation in terms of hyperglycaemia and diabetes.

**Conclusions:**

The intake of calcium, folic acid and magnesium was negatively associated with blood glucose levels in individuals in different quantiles of FPG. Appropriate prevention and treatment strategies should be developed for people with different blood glucose levels.

## Popular scientific summary

- Is the association between mineral and vitamin B group supplementation and blood glucose regulation in normal people real? The effect of mineral and vitamin B group supplementation on continuous blood glucose was evaluated in this study by using quantile regression. Appropriate prevention and treatment strategies should be developed for people with different blood glucose levels.

Hyperglycaemia is a risk factor for infection and is associated with increased mortality during acute illness (1–3). Hyperglycaemia promotes a variety of reactions, including oxidative stress and the formation of advanced glycosylated end products, which have been associated with structural and functional changes in blood vessels that eventually cause dysfunction of several organs, especially the heart, nerves, eyes, and kidneys (4). Fasting plasma glucose (FPG) levels of 7.0 mmol/L or higher were defined as one of the diagnostic criteria for diabetes (5). The global age-standardised diabetes prevalence increased from 4.3% in 1980 to 9.0% in 2014 in men and from 5.0 to 7.9% in women. The number of adults with diabetes in the world increased from 108 million in 1980 to 422 million in 2014 (6). Diabetes is a major cause of mortality, morbidity, and health-system costs worldwide (7, 8).

There is increasing evidence that minerals might play specific roles in hyperglycaemia, the pathogenesis and progression of diabetes, and metabolic complications (9). In pancreatic beta cells, calcium ion influx plays a critical role in regulating glucose-stimulated insulin secretion (GSIS), which is the major mechanism of insulin release (10). Magnesium is an important cofactor in several key enzymes involved in carbohydrate metabolism and is considered to play a role in glucose homeostasis and insulin action (11, 12). Zinc is essential for the formation of both the stored and active forms of insulin and may be involved in the development or progression of both type 1 and type 2 diabetes (13–15). Copper and iron play important roles in the pathological processes that are ongoing in diabetic patients by participating in free radical reactions (16). Additionally, studies have shown that the vitamin B group could play roles in the treatment and prevention of diabetic complications (17). Folic acid supplementation may be associated with glycaemic control in patients with type 2 diabetes mellitus (18). Evidence of vitamin B_1_ and B_12_ deficiencies in individuals with diabetes mellitus has been provided in recent publications (19, 20). Therefore, we wanted to analyse the association of the vitamin B group and mineral intake with blood glucose using quantile regression (QR) analysis. Previous studies have indicated an association between nutrient intake and body index using logistic regression (21). However, the logistic regression requires dichotomizing the response, which implies a huge loss of information on the response variable. Meanwhile, compared with least squares regression and logistic regression, QR makes no assumption of normality and is robust to outliers. Moreover, QR allows for inferences to be made on the entire shape of the distribution and not just on the mean (22).

The development of hyperglycaemia and diabetes is a chronic and continuous process. Many studies have focused on how to regulate blood glucose and complications in diabetic patients with the supplementation of specific nutrients. However, there are few studies on the association between nutrient supplementation and regulating blood glucose in normal people. QR is not limited to explaining the role of certain nutrients in controlling hyperglycaemia but can also be used to explain the role of the intake of specific nutrients in regulating blood glucose in normal people. Therefore, we aimed to mainly investigate the effect of mineral and vitamin B group supplementation on whole continuous blood glucose and observe the effect on different blood glucose populations to achieve the purpose of ‘precise prevention’.

## Materials and methods

**Fig. 1:**
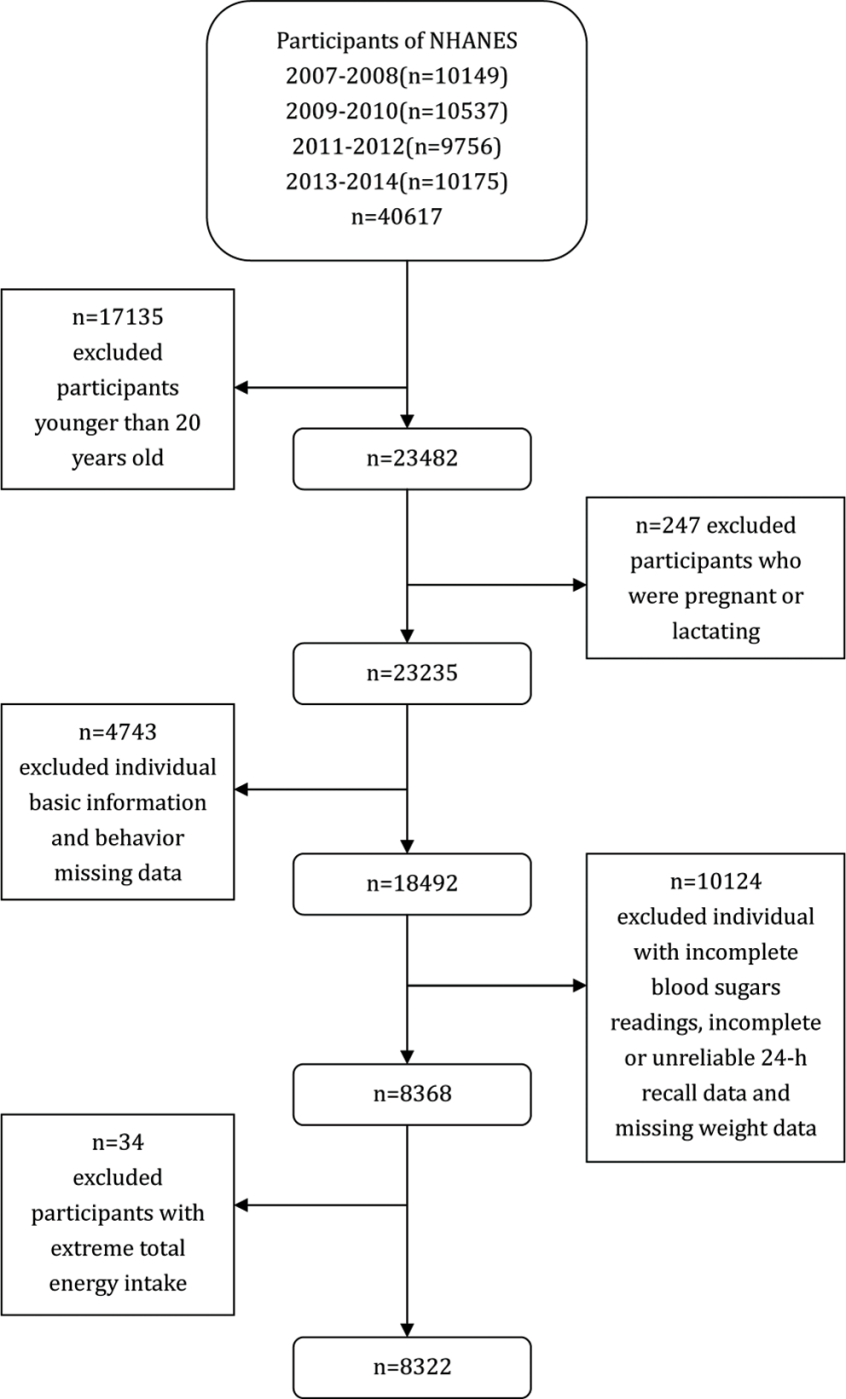
Flow chart of the screening process for the selection of eligible participants (NHANES).

The National Health and Nutrition Examination Survey (NHANES) aims to assess the health and nutritional status of the US population and adopts a complex multi-stage probabilistic sampling design to select representative samples of the civilian non-institutional US population. NHANES participants were first interviewed in their homes and they then participated in a health examination in a mobile examination centre (MEC) (23).The NHANES database is a publicly available dataset for use by researchers around the world; data are released in 2-year cycles and can be downloaded from the NHANES website (24). A total of 40,617 individuals participated in NHANES during the period from 2007 to 2014. We selected 23,482 individuals who were 20 years of age or older. Pregnant or lactating females (*n* = 247) were excluded from the survey. We also excluded individuals with missing basic information and behavioural data (including education (*n* = 34), income (*n* = 2,099), and alcohol consumption missing data (*n* = 2,610). Moreover, individuals who had incomplete blood sugar readings (*n* = 9,878), incomplete or unreliable 24-h recall data (*n* = 245), or missing weight data (*n* = 1) were omitted. Additionally, we excluded individuals who had extreme total energy intakes of <500 or >5,000 kcal/day for females (*n* = 34) and <500 or >8,000 kcal/day for males (*n* = 12). Ultimately, this study included a total of 8,322 participants (4,169 men and 4,153 women) (Fig. 1). As NHANES is a publicly available dataset, the present study was exempt from approval by an institutional review board. All participants provided informed consent before both the interview and examination stages.

According to the NHANES procedures, a fasting glucose blood test was performed on all participants 12 years and older who were examined in the morning session after a 9-h fast. There are seven exclusion criteria, including haemophilia and chemotherapy safety exclusions, fasting <9 h, taking insulin or oral medications for diabetes, refusing phlebotomy, and not drinking the entire Trutol solution within the allotted time. The normal range of FPG is 3.9–6.1 mmol/L (25).

All NHANES participants were eligible for two 24-h dietary recall interviews. The first dietary recall interview was performed in-person in the MEC, and the second interview was conducted over the telephone 3–10 days later. The NHANES dietary data included vitamin B group and mineral variables from the 24-h dietary recall and vitamin B group and mineral variables from the second 24-h recall. We used the average vitamin B group and mineral intake if an individual completed both 24-h recalls. Otherwise, we used the vitamin B group and mineral intake data from the first visit if an individual participated in only one visit. We also assessed the intake of the vitamin B group and minerals from supplements, which was determined using the dietary supplement questionnaire. All participants were asked how often sources of the vitamin B group and minerals were consumed in the last 30 days. We used the average intake of 30 days of dietary supplementation to evaluate the individual dietary supplement level.

In addition to the dietary vitamin B group and mineral intake, we investigated the influence of potential confounding factors, which included age, sex (male and female), race (non-Hispanic white, non-Hispanic black, and other race), educational level (below high school or high school, and above high school), ratio of family income to poverty (≤1, 1–3, >3), smoking status (smoking at least 100 cigarettes in their life or not), and alcohol use (consuming at least 12 alcoholic beverages in 1 year or not).

We used R version 3.5.0 and the package ‘quantreg’ for data analysis (26). Chi-square tests were used to compare the percentages of categorical variables between male and female individuals. Because the variables involved in this study had a skewed distribution, the rank sum test was used to compare the quantitative data between males and females. QR was used to analyse the effects of the vitamin B group and mineral intake on blood glucose levels. QR is a robust approach that does not rely on any assumption of residuals and allows for the estimation of the quantiles of the distribution of the outcome variable (27). Age, income, education, race, smoking and alcohol consumption were included as covariates in the QR.

## Results

Table 1 shows the basic characteristics of the participants; statistically significant differences in race (χ^2^ = 11.549, *P* = 0.016), education (χ^2^ = 6.250, *P* = 0.038), income (χ^2^ = 22.125, *P* = 0.002), smoking (χ^2^ = 129.249, *P* < 0.001), and alcohol consumption (χ^2^ = 340.871, *P* < 0.001) were observed between females and males. Additionally, the vitamin B group and mineral intake and FPG of participants by gender are shown in Table 2 and Supplementary Fig. 1. Moreover, as shown in Table 3, the distribution of blood glucose and quantiles between males and females were different. Therefore, we identified the dietary factors for males and females separately using the QR model.

**Table 1:** Characteristics of participants by gender, NHANES 2007–2014 (*n* = 8,322)

Variable	Male			Female			χ^2^	*P*
	*n*	%*	95% CI	*n*	%	95% CI		
Race							11.549	0.016
Non-Hispanic white	2,019	70.1	(66.6, 73.4)	1,998	70.3	(66.9, 73.5)		
Non-Hispanic black	771	9.1	(7.5, 10.9)	824	10.9	(9.2, 13.0)		
Other	1,379	20.8	(18.1, 23.8)	1,331	18.8	(16.3, 21.5)		
Education							6.250	0.038
<High school	2,082	40.3	(37.0, 43.7)	1,849	37.6	(34.6, 40.7)		
>High school	2,087	59.7	(56.3, 63.0)	2,304	62.4	(59.3, 65.4)		
Income							22.125	0.002
Low income	827	14.8	(12.8, 17.0)	963	17.0	(15.0, 19.2)		
Middle income	1,724	34.4	(32.0, 36.9)	1,743	37.3	(34.4, 40.2)		
High income	1,618	50.8	(47.4, 54.2)	1,447	45.7	(42.3, 49.2)		
Smoking							129.249	<0.001
Yes	2,244	51.8	(48.9, 54.6)	1,548	39.3	(36.8, 42.0)		
No	1,925	48.2	(45.4, 51.1)	2,605	60.7	(58.0, 63.2)		
Alcohol consumption								
Yes	3,482	86.6	(84.7, 88.3)	2,607	69.9	(67.0, 72.6)	340.871	<0.001
No	687	13.4	(11.7, 15.3)	1,546	30.1	(27.4, 33.0)		

* All data are weighted to be nationally representative.

**Table 2:** The vitamin B group and mineral intake, and fasting plasma glucose (FPG) of participants by gender, NHANES 2007–2014 (*n* = 8,322) [mean (95% CI)]*

	Male	Female
Age (years)	46.49 (45.56, 47.43)	48.61 (47.79, 49.44)
FPG (mg/dL)	108.02 (106.53, 109.50)	102.89 (101.85, 103.92)
FPG (mmol/L)	5.99 (5.91, 6.08)	5.71 (5.65, 5.77)
Calcium (mg)	1221.46 (1186.01, 1256.90)	1105.85 (1074.03, 1137.67)
Magnesium (mg)	372.71 (362.48, 382.94)	305.34 (294.20, 316.48)
Phosphorus (mg)	1633.50 (1601.75, 1665.26)	1204.62 (1180.27,1228.97)
Iron (mg)	19.44 (18.94, 19.94)	17.78 (17.16, 18.40)
Zinc (mg)	17.98 (17.32, 18.64)	14.74 (14.22, 15.26)
Copper (mg)	1.79 (1.72, 1.86)	1.53 (1.48, 1.59)
Sodium (mg)	4119.64 (4048.80,4190.48)	3015.94(2962.99,3068.89)
Potassium (mg)	3097.06(3038.39,3155.73)	2413.20(2360.91,2465.49)
Selenium (mcg)	154.27 (150.16, 158.39)	110.62 (107.82, 113.42)
Thiamine (VB_1_) (mg)	4.97 (4.39, 5.56)	5.70 (4.33, 7.06)
Riboflavin(VB_2_) (mg)	4.66 (4.25, 5.06)	5.25 (4.10, 6.41)
Niacin (VB_3_) (mg)	44.65 (39.27, 50.04)	34.03 (31.20, 36.85)
Pyridoxine (VB_6_) (mg)	5.53 (4.96, 6.10)	6.55 (4.78, 8.33)
Folate (VB_9_) (mcg)	821.68 (794.37, 848.99)	735.09 (712.14, 758.04)
Cobalamin (VB_12_) (mcg)	44.58 (33.55, 55.61)	70.97 (52.45, 89.50)

* All data are weighted to be nationally representative.

**Table 3:** The distribution of fasting plasma glucose (FPG)* and quantiles by gender

	0.1	0.2	0.3	0.4	0.5	0.6	0.7	0.8	0.9
Male									
FPG (mmol/L)	5.00	5.22	5.38	5.50	5.66	5.83	6.11	6.50	7.55
FPG (mg/dL)	90.00	94.00	97.00	99.00	102.00	105.00	110.00	117.00	136.00
Female									
FPG (mmol/L)	4.72	4.94	5.11	5.27	5.44	5.61	5.83	6.22	7.05
FPG (mg/dL)	85.00	89.00	92.00	95.00	98.00	101.00	105.00	112.00	127.00

* The normal range of FPG is 3.9–6.1 mmol/L (70.2–109.8 mg/dL).

After adjusting for age, income, education, race, smoking, and alcohol consumption in males, we found that folic acid intake showed a negative association with blood glucose levels in both normal and hyperglycaemic individuals (5.38–6.50 mmol/L). The intake of calcium was negatively associated with blood glucose levels in normal individuals (5.38–5.83 mmol/L). Additionally, the intake of magnesium was negatively associated with blood glucose levels in the entire conditional blood glucose distribution (Table 4). In addition, the association of other mineral and vitamin B group intake with blood glucose in males is shown in Supplementary Tables 1 and 3.

**Table 4:** Quantile regression coefficients between fasting plasma glucose and the intake of folic acid, calcium, and magnesium for males and females^#^

	0.1	0.2	0.3	0.4	0.5	0.6	0.7	0.8	0.9
Male									
Folic acid (mg)	−1.407	−1.102	−1.091*	−0.980*	−0.609*	−0.359*	−0.616*	−1.023*	−1.732
	(−3.311, 0.121)	(−2.850, 0.093)	(−2.404, −0.315)	(−2.196, −0.053)	(−2.001, −0.100)	(−2.200, −0.220)	(−1.757, −0.397)	(−1.860, −0.188)	(−2.145, 1.163)
Calcium (g)	−0.491	−0.702	−0.903*	−0.692*	−0.979*	−0.883*	−0.641	−0.169	−0.718
	(−1.453, 0.505)	(−1.270, 0.093)	(−1.703, −0.180)	(−1.574, −0.290)	(−1.661, −0.055)	(−2.233, −0.078)	(−1.606, 0.618)	(−2.234, 1.543)	(−2.132, 3.074)
Magnesium (g)	−4.165*	−4.926*	−5.093*	−4.670*	−3.387*	−1.803*	−3.473*	−4.036*	−5.208*
	(−5.612, −0.411)	(−8.654, −1.038)	(−8.407, −1.436)	(−7.303, −0.611)	(−6.739, −0.480)	(−7.486, −0.182)	(−7.141, −1.137)	(−6.633, −2.231)	(−11.844, −2.033)
Female									
Folic acid (mg)	−1.657*	−1.523*	−1.772*	−0.970*	−1.485*	−1.593*	−2.128*	−1.508	−1.991
	(−2.233, −0.262)	(−2.901, −0.324)	(−2.585, −0.559)	(−2.278, −0.268)	(−2.177, −0.824)	(−2.162, −0.860)	(−2.999, −0.578)	(−3.846, 0.066)	(−4.174, 0.146)
Calcium (g)	−1.400*	−1.073*	−1.594*	−1.124*	−1.401*	−1.080*	−1.431*	−1.344	−0.171
	(−1.950, −0.376)	(−2.106, −0.502)	(−2.316, −0.958)	(−2.296, −0.496)	(−2.340, −0.533)	(−1.877, −0.241)	(−2.245, −0.237)	(−2.995, 0.555)	(−3.476, 1.903)
Magnesium (g)	−2.364	−2.619	−4.300*	−3.244*	−3.363	−2.141*	−3.983*	−7.295*	−9.674*
	(−7.952, 1.410)	(−5.680, 0.589)	(−7.034, −0.739)	(−6.630, −0.913)	(−6.814, 0.331)	(−5.627, −0.214)	(−8.388, −0.616)	(−11.42, −1.695)	(−16.319, −0.700)

# Adjusted for age, income, education, race, smoking, and alcohol consumption.

* *P* < 0.05.

Similar to males, after adjusting for age, income, education, race, smoking, and alcohol consumption, we found that folic acid and calcium intake showed a negative association with blood glucose levels in normal female individuals (<5.83 mmol/L). The intake of magnesium was negatively associated with blood glucose levels in most of the quantiles (Table 4). In addition, the association of other mineral and vitamin B group intake with blood glucose in females is shown in Supplementary Tables 2 and 4.

**Fig 2:**
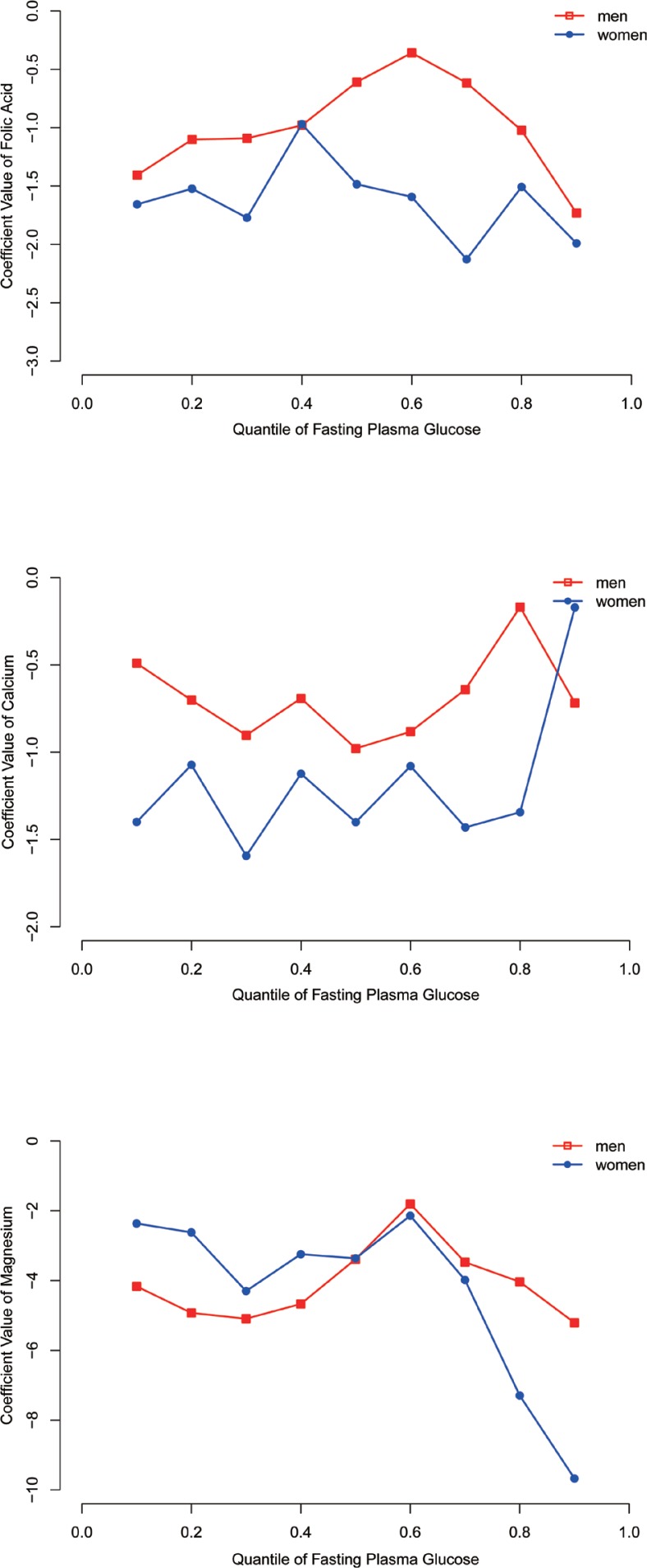
Association between FPG and the intake of folic acid, calcium and magnesium for male and female.

We found that folic acid intake was more effective in regulating blood glucose levels in females. Similar results were observed for calcium intake. Moreover, the intake of magnesium may be more effective for regulating blood glucose levels in females with hyperglycaemia. However, this finding requires additional statistical analysis and validation (Fig. 2). The association between FPG and the intake of other minerals and vitamin B groups for males and females is shown in Supplementary Fig. 2. Moreover, the association between the risk of hyperglycemia and the intake of folic acid, calcium, and magnesium for males and female has been shown in Supplementary Fig. 3.

## Discussion

Hyperglycaemia and diabetes are currently becoming popular research topics. However, little is known about how the whole continuum of blood glucose is associated with commonly researched nutrient supplementation in terms of hyperglycaemia and diabetes. Therefore, we used the QR model to analyse the effect of mineral and group B vitamin supplementation on the entire conditional distribution of blood glucose. Our study revealed that the intake of calcium, folic acid, and magnesium was negatively associated with blood glucose levels in some quantiles after adjusting for age, income, education, race, smoking, and alcohol consumption in males and females.

Folic acid is a promising agent for the prevention and treatment of hyperglycaemia and diabetes by reducing homocysteine and regulating insulin resistance and glycaemia (28). Our study also revealed that folic acid intake could play a role in regulating blood glucose levels in hyperglycaemic male individuals, which was consistent with the findings of Gargari’s study (29). This finding indicated that folic acid was not only useful as a dietary supplementation for pregnant women to provide protection against neural tube defects (30) but was also effective as a medicine for men with hyperglycaemia and diabetes as it helped in effectively regulating blood glucose levels, which is a popular research topic. The meta-analysis has shown that clinical folic acid supplementation is beneficial for insulin resistance and glycaemic control. However, there is a lack of explicit guidance on intervention time and dose (28). Relevant clinical trials should be designed to explore the clinical folic acid supplementation time and specific dose in the future. Specifically, our study revealed that folic acid intake showed a negative association with blood glucose levels in males and females with normal blood glucose levels. Hence, our findings suggest that the intake of folic acid in healthy people could prevent hyperglycaemia, thus changing our preventive strategy from a high-risk strategy to a population-wide strategy, which is a first-level prevention strategy oriented by public health thinking.

A potentially important role for calcium in the development of diabetes has been suggested by case-control studies in which calcium intake was found to be lower in patients with diabetes than in controls (31). Additionally, previous studies have shown that calcium channels play a role in β-cell thioredoxin-interacting protein (TXNIP) expression and β-cell apoptosis, thus enhancing endogenous insulin levels (32). However, the results of epidemiological studies are inconsistent. Some studies suggested a beneficial role of calcium intake in reducing the risk of type 2 diabetes (33, 34). Conversely, Gagnon’s study indicated that dietary calcium intake was not associated with a reduced risk of diabetes (35). Based on a meta-analysis, the effects of calcium supplementation as a component of dairy products in relation to glycaemia or insulin resistance have shown conflicting results (36). Moreover, there have been few studies on the direct role of calcium in the mechanism underlying the regulation of blood glucose. Therefore, the association of calcium intake with blood glucose is still controversial. Our study actually indicated that calcium intake was not related to blood glucose regulation in hyperglycaemic people using QR. Our results also showed that the intake of calcium contributes to maintaining healthy blood glucose levels. The role of genetic factors in the development of diabetes is universally known (37). Therefore, for some people with normal blood glucose and family history of diabetes, we consider that calcium supplementation may appropriately reduce their risk of diabetes. Meanwhile, a great deal of work is needed to explore the possible mechanism underlying the association. Then, we can propose different prevention strategies and truly achieve precise prevention.

Many meta-analyses have shown a significant inverse association between magnesium intake and blood glucose. Magnesium intake may decrease the risk of diabetes (38–41). Reduced magnesium intake and augmented magnesium urinary loss cause magnesium deficiency in diabetics, which is very common (11, 42, 43). Magnesium could regulate insulin sensitivity via its involvement in the regulation of insulin signalling, the phosphorylation of insulin receptor kinase, the post-receptorial action of insulin, and insulin-mediated cellular glucose uptake, thereby regulating blood glucose (12, 43).Dietary magnesium deficiency may cause insulin resistance, as shown by several studies both in humans and in experimental animals (44).The intake of magnesium has been shown to improve FPG levels and insulin sensitivity in hyperglycaemic subjects (45, 46) and to improve insulin sensitivity in healthy subjects (47); these findings are consistent with our research results. However, new intervention studies are needed to elucidate the standardization of the type, dose, and time of magnesium supplementation, thus making robust guidelines for clinical practice.

However, our study also has some limitations. First, we cannot infer causal interpretations of the relationship of the vitamin B group and mineral intake with hyperglycaemia or diabetes risks because of the cross-sectional design of the study. Second, we used the two 24-h diet recalls to reflect the individuals’ usual intake, but this method maybe affected by recall bias. Moreover, we cannot infer the mechanisms behind the reverse association of the vitamin B group and mineral intake with blood glucose due to this being an epidemiological study. A great deal of work is needed to systematically analyse the mechanisms and effects of the vitamin B group and mineral intake on blood glucose regulation.

## Conclusion

The effects of the vitamin B group and mineral intake are different in different quantiles of FPG. FPG had a negative association with folic acid in individuals with normal and high FPG, with calcium in individuals with normal FPG, and with magnesium in all FPG quantiles for males. FPG had a negative association with folic acid and calcium in individuals with normal FPG and with magnesium in most of the quantiles for females. Our study suggests that appropriate prevention and treatment strategies should be developed for people with different blood glucose levels.

## Conflict of interest and funding

The authors declare that there are no conflicts of interest regarding the publication of this article. The authors have not received any funding or benefits from industry or elsewhere to conduct this study.

## Supplementary Material

Association of multiple mineral and vitamin B group intake with blood glucose using quantile regression analysis: NHANES 2007–2014
