# Supplementary material for: Association of multiple mineral and vitamin B group intake with blood glucose using quantile regression analysis: NHANES 2007–2014
Source: Food Nutr Res. 2019 Dec 3;63:10.29219/fnr.v63.3560. doi: 10.29219/fnr.v63.3560 (PMC6958618; doi:10.29219/fnr.v63.3560)
Supplement: Association of multiple mineral and vitamin B group intake with blood glucose using quantile regression analysis: NHANES 2007–2014 [file FNR-63-3560-s001.pdf]

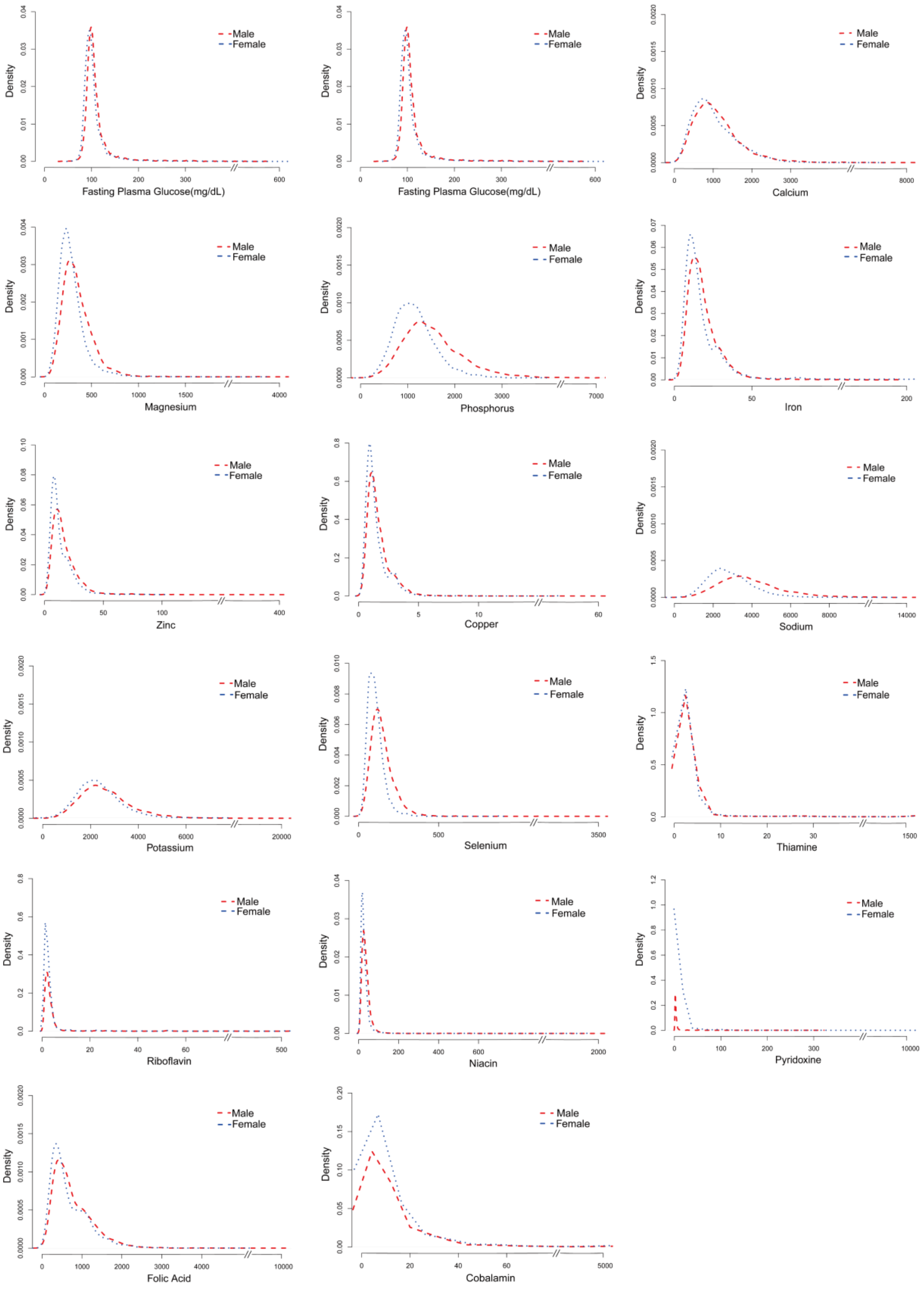

Fig S1 Density plot of the vitamin B group and mineral intake, and FPG of participants by gender.

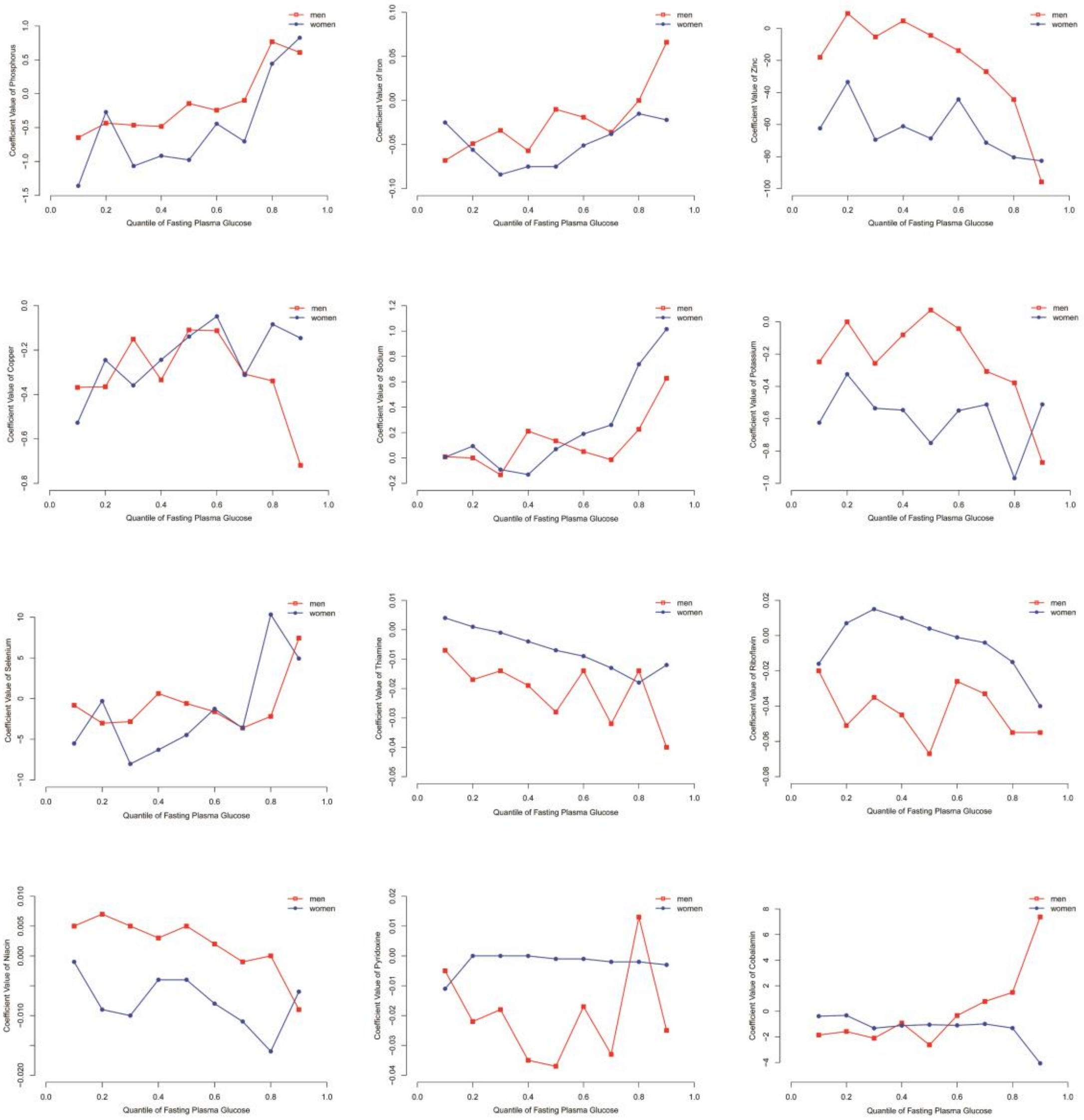

Fig S2 Association between FPG and the intake of other mineral and vitamin B group for male and female.

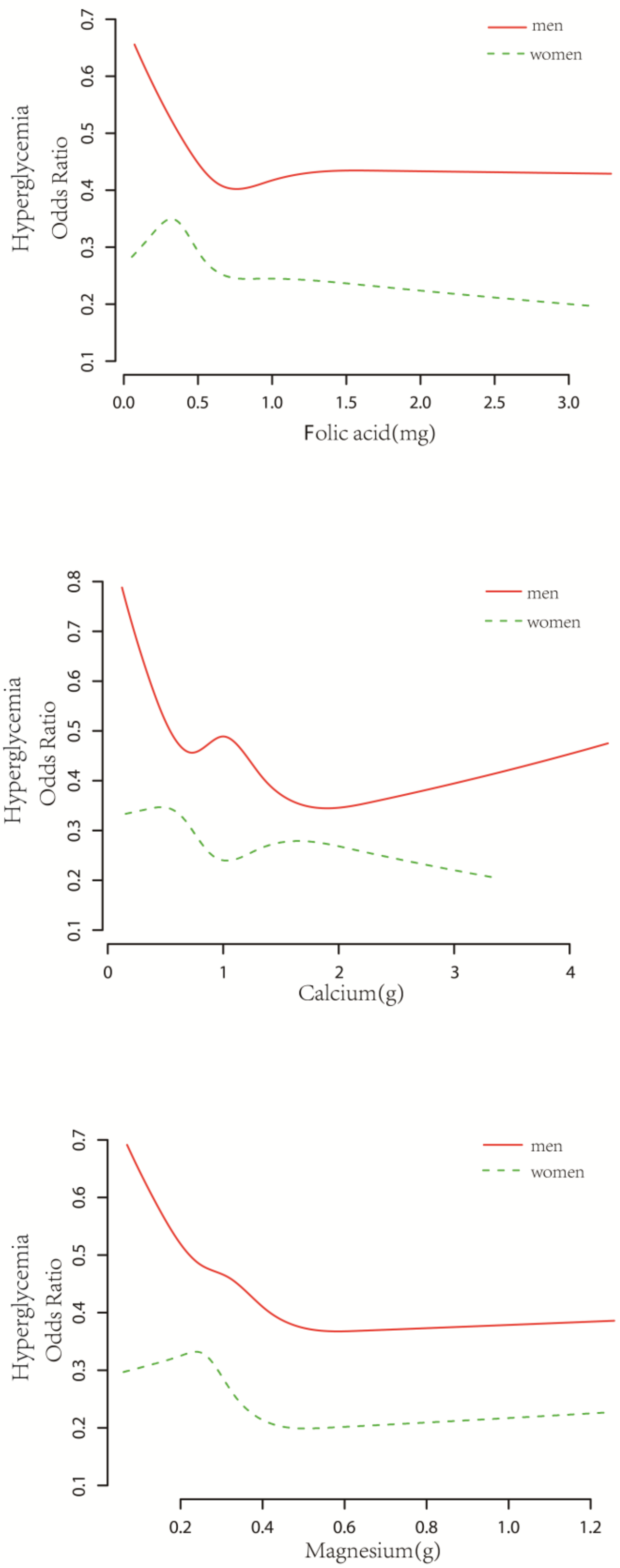

Figure S3 Association between the risk of hyperglycemia and the intake of folic acid, calcium and magnesium for male and female.

Table S1 QR coefficients between FPG and other B vitamin group intake for males#.

	0.1	0.2	0.3	0.4	0.5	0.6	0.7	0.8	0.9
Thiamine(VB_1)(mg)	-0.007	-0.017	-0.014	-0.019*	-0.028	-0.014*	-0.032	-0.014	-0.040
	(-0.085,0.000)	(-0.071,0.001)	(-0.063,0.003)	(-0.051,-0.006)	(-0.058,0.016)	(-0.054,-0.004)	(-0.048,0.043)	(-0.063,0.069)	(-0.076,0.097)
Riboflavin(VB_2)(mg)	-0.020*	-0.051	-0.035	-0.045*	-0.067	-0.026	-0.033	-0.055	-0.055
	(-0.147,-0.002)	(-0.103,0.007)	(-0.114,0.001)	(-0.087,-0.025)	(-0.095,0.025)	(-0.121,0.010)	(-0.119,0.041)	(-0.152,0.055)	(-0.120,0.172)
Niacin (VB_3) (mg)	0.005	0.007	0.005	0.003	0.005*	0.002	-0.001	0.000	-0.009
	(-0.107,0.009)	(-0.063,0.013)	(-0.020,0.012)	(-0.007,0.010)	(0.001,0.009)	(0.000,0.007)	(-0.002,0.004)	(-0.004,0.029)	(-0.011,0.370)
Pyridoxine (VB_6) (mg)	-0.005	-0.022	-0.018*	-0.035*	-0.037	-0.017	-0.033	0.013	-0.025
	(-0.123,0.018)	(-0.097,0.009)	(-0.073,-0.004)	(-0.054,-0.002)	(-0.067,0.028)	(-0.078,0.017)	(-0.073,0.028)	(-0.085,0.023)	(-0.044,0.058)
Cobalamin(VB_{12}) (mcg)	-1.857	-1.581*	-2.107	-0.913	-2.627	-0.330	0.772	1.478	7.385
	(-5.112,0.697)	(-5.762,-0.789)	(-6.143,1.756)	(-5.245,1.039)	(-5.083,4.257)	(-5.978,3.608)	(-5.814,8.863)	(-2.957,19.362)	(-4.607,62.893)

#Adjusted for age, income, education, race, smoking and alcohol consumption.

*p<0.05.

Table S2 QR coefficients between FPG and other B vitamin group intake for females#

	0.1	0.2	0.3	0.4	0.5	0.6	0.7	0.8	0.9
Thiamine(VB_1)(mg)	0.004	0.001	-0.001	-0.004	-0.007	-0.009	-0.013	-0.018	-0.012
	(-0.116,0.004)	(-0.034,0.007)	(-0.034,0.020)	(-0.027,0.012)	(-0.037,0.017)	(-0.038,0.023)	(-0.044,0.063)	(-0.019,0.063)	(-0.032,0.052)
Riboflavin(VB_2)(mg)	-0.016	0.007	0.015	0.010	0.004	-0.001	-0.004*	-0.015	-0.040
	(-0.692,0.036)	(-0.080,0.025)	(-0.078,0.019)	(-0.042,0.015)	(-0.051,0.007)	(-0.047,0.001)	(-0.057,-0.004)	(-0.067,0.106)	(-0.040,0.178)
Niacin (VB_3) (mg)	-0.001	-0.009	-0.010	-0.004	-0.004	-0.008	-0.011	-0.016	-0.006*
	(-0.071,0.001)	(-0.025,0.011)	(-0.015,0.006)	(-0.019,0.008)	(-0.023,0.002)	(-0.023,0.002)	(-0.023,0.001)	(-0.024,0.012)	(-0.032,-0.001)
Pyridoxine (VB_6) (mg)	-0.011	0.000	0.000	0.000	-0.001*	-0.001*	-0.002	-0.002	-0.003
	(-0.933,0.000)	(-0.098,0.000)	(-0.077,0.000)	(-0.044,0.000)	(-0.045,-0.001)	(-0.045,-0.001)	(-0.051,0.014)	(-0.045,0.106)	(-0.003,1.298)
Cobalamin(VB_{12}) (mcg)	-0.380	-0.318	-1.324	-1.118	-1.047	-1.099	-0.987	-1.315	-4.069
	(-6.245,1.130)	(-2.208,1.406)	(-3.220,0.901)	(-3.873,0.939)	(-4.158,0.955)	(-3.088,0.401)	(-3.673,0.032)	(-4.752,3.731)	(-4.447,7.963)

#Adjusted for age, income, education, race, smoking and alcohol consumption.

*p<0.05.

Table S3 QR coefficients between FPG and other mineral intake for males#

	0.1	0.2	0.3	0.4	0.5	0.6	0.7	0.8	0.9
Phosphorus(g)	-0.647	-0.434	-0.464	-0.482	-0.145	-0.244	-0.099	0.766	0.608
	(-1.189,0.812)	(-1.152,0.216)	(-1.502,0.034)	(-1.183,0.758)	(-1.213,0.725)	(-1.210,1.140)	(-1.260,1.573)	(-0.519,2.021)	(-1.361,3.924)
Iron (mg)	-0.068*	-0.049*	-0.034	-0.057*	-0.010	-0.019	-0.036	0.000	0.066
	(-0.102,-0.011)	(-0.085,-0.004)	(-0.101,0.001)	(-0.080,-0.005)	(-0.080,0.030)	(-0.077,0.011)	(-0.080,0.030)	(-0.080,0.121)	(-0.147,0.248)
Zinc (g)	-18.039	9.291	-5.336	4.609	-4.429	-13.901	-27.058	-44.430	-95.778
	(-98.074,30.100)	(-67.194,20.776)	(-34.540,11.819)	(-48.768,15.577)	(-18.187,21.838)	(-33.378,21.597)	(-45.616,37.699)	(-65.512,46.152)	(-97.878,76.240)
Copper(mg)	-0.368	-0.365	-0.151	-0.334	-0.109	-0.113	-0.308*	-0.339*	-0.719
	(-1.207,0.228)	(-1.188,0.155)	(-1.064,0.089)	(-0.877,0.035)	(-0.960,0.033)	(-0.834,0.074)	(-0.640,-0.201)	(-0.839,-0.261)	(-1.160,0.017)
Sodium (g)	0.009	0.000	-0.133	0.211	0.134	0.050	-0.014	0.225	0.628
	(-0.468,0.372)	(-0.263,0.247)	(-0.380,0.252)	(-0.326,0.485)	(-0.230,0.469)	(-0.368,0.443)	(-0.291,0.490)	(-0.240,0.680)	(-0.342,2.303)
Potassium (g)	-0.248	0.000	-0.257	-0.081	0.073	-0.043	-0.307	-0.378	-0.871
	(-0.825,0.469)	(-0.491,0.272)	(-0.721,0.291)	(-0.429,0.465)	(-0.479,0.395)	(-0.592,0.337)	(-0.735,0.246)	(-0.808,0.326)	(-1.628,0.257)
Selenium (mg)	-0.821	-3.018	-2.827	0.627	-0.598	-1.609	-3.604	-2.185	7.435
	(-9.572,4.165)	(-12.484,2.798)	(-10.296,1.602)	(-7.014,4.488)	(-2.970,6.314)	(-3.450,6.736)	(-6.143,9.902)	(-6.099,15.948)	(-12.927,48.773)

#Adjusted for age, income, education, race, smoking and alcohol consumption.

*$p<0.05$.

Table S4 QR coefficients between FPG and other mineral intake for females#

	0.1	0.2	0.3	0.4	0.5	0.6	0.7	0.8	0.9
Phosphorus(g)	-1.358	-0.271	-1.066*	-0.916	-0.976	-0.443	-0.703	0.442	0.825
	(-2.511,0.252)	(-1.409,0.640)	(-1.960,-0.024)	(-1.745,0.034)	(-2.051,0.179)	(-1.584,0.490)	(-1.903,0.900)	(-2.064,3.174)	(-1.920,3.735)
Iron (mg)	-0.025	-0.056	-0.084*	-0.075	-0.075*	-0.051	-0.038	-0.015	-0.022
	(-0.084,0.010)	(-0.067,0.027)	(-0.094,-0.041)	(-0.118,0.039)	(-0.111,-0.035)	(-0.100,0.010)	(-0.084,0.020)	(-0.082,0.047)	(-0.068,0.131)
Zinc (g)	-62.351*	-33.482	-69.468*	-61.087*	-68.672	-44.321	-71.312*	-80.464	-82.616
	(-83.498,-25.067)	(-97.716,0.743)	(-103.583,-43.692)	(-104.757,-25.960)	(-93.797,3.408)	(-100.158,16.794)	(-120.308,-9.351)	(-200.396,20.138)	(-255.340,30.955)
Copper(mg)	-0.527	-0.245	-0.359	-0.244	-0.139	-0.048	-0.312	-0.084	-0.146
	(-1.411,0.168)	(-0.674,0.242)	(-0.874,0.034)	(-0.737,0.151)	(-0.712,0.249)	(-0.527,0.482)	(-0.796,0.439)	(-1.222,1.260)	(-1.225,1.708)
Sodium (g)	0.006	0.093	-0.092	-0.131	0.069	0.189	0.260	0.738	1.015
	(-0.656,0.417)	(-0.229,0.439)	(-0.413,0.260)	(-0.473,0.348)	(-0.372,0.650)	(-0.183,0.603)	(-0.306,0.999)	(-0.182,1.318)	(-0.212,2.704)
Potassium (g)	-0.626	-0.326	-0.537*	-0.548*	-0.752	-0.551	-0.514	-0.970	-0.513
	(-1.162,0.073)	(-1.217,0.138)	(-0.949,-0.109)	(-1.071,-0.096)	(-1.134,0.042)	(-0.938,0.022)	(-1.236,0.069)	(-1.801,0.234)	(-1.663,0.984)
Selenium (mg)	-5.512	-0.305	-8.027	-6.297	-4.472	-1.261	-3.629	10.334	4.934
	(-14.036,5.361)	(-5.647,7.857)	(-14.749,1.502)	(-14.880,2.257)	(-15.075,7.098)	(-9.052,6.811)	(-12.259,13.145)	(-15.636,36.920)	(-14.238,39.145)

#Adjusted for age, income, education, race, smoking and alcohol consumption.
*p<0.05.
